# How Local Order Leads to Shape Selectivity in Disordered Materials: The Case of FAU-FER Interzeolite Transformation Intermediates

**DOI:** 10.1021/acscatal.4c07182

**Published:** 2025-03-04

**Authors:** Julia Telles de Souza, Alexandre Ferreira Young, Eduardo F. Sousa-Aguiar, Pedro N. Romano, Javier García-Martínez, João M. A. R. De Almeida

**Affiliations:** † Escola de Química, Universidade Federal do Rio de Janeiro, Av. Athos da Silveira Ramos, 149, Rio de Janeiro 21941-909, Brazil; ‡ LIPCAT (Laboratório de Intensificação de Processos e Catálise), Universidade Federal do Rio de Janeiro (UFRJ), Rio de Janeiro 21941-594, Brazil; § Campus Duque de Caxias, Universidade Federal do Rio de Janeiro, Rodovia Washington Luiz, 19593, Rio de Janeiro 25240-005, Brazil; ∥ Nanotechnology Engineering Program, Alberto Luiz Coimbra Institute for Graduate Studies and Research in Engineering (COPPE), Federal University of Rio de Janeiro, Avenida Horacio Macedo, 2030, Rio de Janeiro 21941-972, Brazil; ⊥ Laboratorio de Nanotecnología Molecular, Departamento de Química Inorgánica, Universidad de Alicante, Alicante 03690, Spain; # Instituto de Química, Universidade Federal do Rio de Janeiro, Av. Athos da Silveira Ramos, 149, Rio de Janeiro 21941-909, Brazil; ¶ PGQu (Programa de Pós-Graduação em Química), Universidade Federal do Rio de Janeiro, Av. Athos da Silveira Ramos, 149, Rio de Janeiro 21941-909, Brazil

**Keywords:** ferrierite (FER) zeolite, shape selectivity, interzeolite transformation intermediates, microwave, DME, acid catalysis

## Abstract

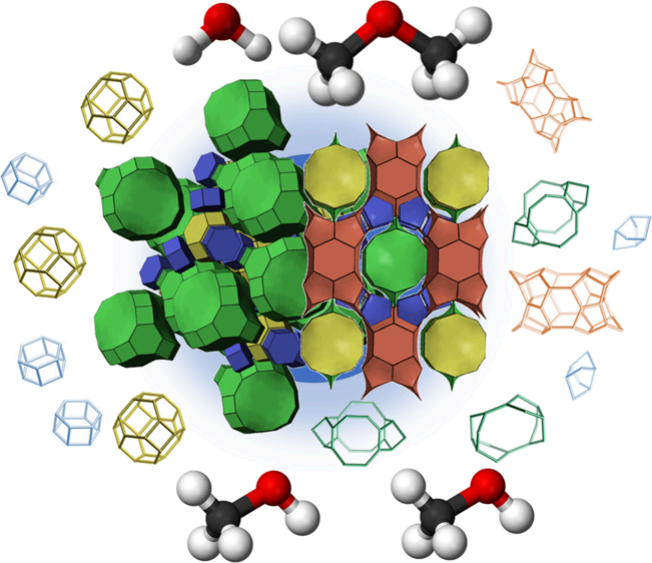

This study presents a series of Interzeolite Transformation Intermediates (ITIs) derived from FAU-to-FER interconversion. These hybrid materials, obtained through precise control of the interconversion process, exhibit both large mesoporosity and FER topology-type pore confinement, resulting in high conversion and remarkable shape selectivity despite their disordered structure at the long range. We demonstrated this unique combination of properties in three different catalytic tests. The local order within these ITIs is sufficient to create pore confinement, which not only produces remarkable shape selectivity but also enhances conversion by increasing accessibility. Specifically, the ITIs show a 10-fold increase in activity for Friedel–Crafts alkylation, a 16-fold increase in activity for triisopropylbenzene (TiPBz) cracking, and a two-fold increase in methanol dehydration to dimethyl ether (DME) compared to commercial ferrierite all while maintaining the selectivity of FER. These results highlight the potential of FAU-to-FER ITIs as high-performance catalysts that combine the accessibility of disordered structures with the selectivity typically associated with well-ordered zeolites, opening avenues in zeolite-based catalysis.

## 1 Introduction

Zeolites, characterized by their microporous aluminosilicate structures, are integral to numerous applications in catalysis, ion exchange, and separation processes.^1,2^ Ferrierite (FER), a member of the zeolite family, is particularly noted for its unique two-dimensional channel system that facilitates its use in various catalytic processes including the production of dimethyl ether (DME) from methanol^3,4^ and the methanol to olefins (MTO) process.^3,5,6^ Despite its utility, the narrow pore structure of FER leads to diffusion limitations, which hinder its use for several other applications.^7−11^

To address these limitations, significant research has focused on enhancing the accessibility of zeolites without compromising their inherent shape selectivity.^12−16^ Approaches such as introducing hierarchical porosity or modifying the synthesis conditions have shown promise in achieving this balance.^17,18^ For example, the incorporation of mesopores into zeolitic frameworks has been demonstrated to improve the diffusion of bulky reactants, thereby expanding the utility of these materials in industrial applications.^19,20^

Alternatively, poorly crystalline zeolites, such as embryonic zeolites, can exhibit short-range order structures that contribute to shape selectivity. These materials preserve certain local structural characteristics of crystalline zeolites, such as pore systems with well-defined dimensions as well as cages and channels that align with the organic structure-directing agents (OSDAs). This short-range order creates a network of pores and cavities that can discriminate between molecules based on size and shape, even in the absence of long-range crystallinity. Local order in disordered catalysts can provide significant control over the reaction pathways and product distributions.^21,22^

A promising strategy to provide this local order without the inherent trade-offs of working with nanosized zeolites is partial interzeolite transformation. The García-Martínez group recently demonstrated that ITIs from FAU-to-BEA^23^ and FAU-to-MFI^24^ exhibited remarkable catalytic performance in the conversion of bulky molecules due to mesoporosity generated during the interconversion process.

In this study, we introduce the first example of an interzeolite transformation intermediates (ITIs) derived from the FAU-to-FER interconversion. By halting the interconversion process at strategic points, we synthesized ITIs that demonstrate significant shape selectivity, despite their seemingly disordered structures. This novel class of hybrid materials has been achieved through precise control of the interconversion conditions, exhibits a unique combination of accessibility and catalytic selectivity.

Our findings, supported by data from three distinct catalytic tests, reveal that these ITIs can significantly outperform commercial ferrierite. The ITIs show a 10-fold increase in activity for Friedel–Crafts alkylation, a 16-fold increase in activity for triisopropylbenzene (TiPBz) cracking, and a 2-fold increase in methanol dehydration to DME. Importantly, these enhancements are achieved without sacrificing the inherent selectivity features of FER, demonstrating the ITIs’ ability to merge the accessibility of disordered structures with the selectivity typically associated with well-ordered zeolites.^26^

These results highlight the potential of FAU-to-FER ITIs as high-performance catalysts that can effectively bridge the gap between the accessibility required for handling large molecules and the shape selectivity necessary for efficient catalysis. This study not only advances our understanding of zeolite functionality but also opens new avenues for the application of zeolites in industrial catalysis.

## 2 Materials and Methods

### 2.1 Materials

Pyrrolidine (99%, Sigma-Aldrich), sodium silicate solution (SiO_2_ 26.5%, Na_2_O 10.6%, Sigma-Aldrich), deionized water, NH_4_Y zeolite (Zeolyst International, SiO_2_/Al_2_O_3_ of 12, CBV712), and ammonium chloride (P.A, Isofar) were employed in the zeolitic interconversion synthesis. Commercial ferrierite (H-FER, Zeolyst International, CP914C) served as the reference material. For the catalyst test, mesitylene (98%, Sigma-Aldrich) and benzyl alcohol (anhydrous, 99.8%, Sigma-Aldrich) were utilized.

### 2.2 Interzeolite Transformation FAU-FER

The synthesis process, adapted from the one reported by Bolshakov et al.,^25^ started by dissolving pyrrolidine (Py) in deionized water at room temperature, followed by the addition of sodium silicate solution and zeolite Y. The molar composition used was 1 Py:1.82 Na_2_O:0.42 Al_2_O_3_:4.80 SiO_2_:215 H_2_O. The mixture was vigorously stirred at room temperature for 4 h. Subsequently, 50 mL of the mixture was transferred to a Microwave Flexi Wave Teflon-coated reactor which was set to a temperature of 140 °C. Interconversions were carried out at different crystallization times, namely, 24, 36, 48, 52, 56, 60, and 72 h. These times were used to name the samples that were prepared. After the specified crystallization times, the solids were filtered, washed with deionized water until pH ∼ 7–8, and then dried at 100 °C for 12 h. Finally, the samples were calcined in air at 550 °C (1 °C/min) for 7 h. To produce the acid form of these materials, the samples were ion-exchanged in an aqueous 1 M NH_4_Cl solution at 80 °C for 24 h. The resulting solids were then separated via filtration and calcined in air at 550 °C (1 °C/min) for 5 h to remove NH_3_.

### 2.3 Catalytic Tests

#### 2.3.1 Fridel–Crafts Alkylation

Based on the procedure described by Jain et al.,^27^ mesitylene and benzyl alcohol were employed as reagents for the Friedel–Crafts alkylation reaction. The reaction took place in a 50 mL round-bottomed flask. More specifically, 100 mg of catalysts was added to 95 mmol of mesitylene, and the mixture was heated to 120 °C in an oil bath. Subsequently, 1 mmol of benzyl alcohol was introduced into the mixture. Samples were systematically collected at intervals (0, 1, 2, 4, 6, and 8 h) to evaluate the reaction kinetics. The collected aliquots underwent analysis by using gas chromatography with a flame ionization detector. Considering the excess of mesitylene, the reaction samples were evaluated for benzyl alcohol conversion and the selectivities toward 1,3,5-trimethyl-2-benzylbenzene (TM2B) and dibenzyl ether (DBE), as illustrated in Figure 3a. Selectivities were measured at a constant isoconversion of 10%.

#### 2.3.2 Cracking of Triisopropylbenzene (TiPBz)

The triisopropylbenzene (TiPBz) cracking was monitored based on the protocol described by Abdulridha et al.^28^ More specifically, 10 mg of zeolite was placed in a borosilicate glass liner (Restek 20793, with an internal diameter of 4 nm, an external diameter of 6.3 nm, and a length of 72 nm) between two beds of quartz wool (Shimadzu). The zeolite was initially calcined at 320 °C for 2 h to remove the inherited moisture in zeolites. Then, 2 μL of TiPBz was injected, vaporized, and transported to the bed by a helium gas flow of 200 mL·min^–1^. A portion of the products was directed to the column (DB-WAX, internal diameter = 0.20 mm, length = 50 m, film thickness = 0.2 μm, Agilent) at a 200:1 split ratio and analyzed using a flame ionization detector (FID) at 300 °C. The column temperature started at 80 °C, increased gradually to 220 °C at a rate of 10 °C·min^–1^, and was held at this temperature for 10 min. This procedure was repeated 30 times for each sample. For this purpose, we used a pulse technique with gas chromatography (GC Thermo Trace 1610).

#### 2.3.3 Methanol to Dimethyl Ether

The catalytic activity for dimethyl ether (DME) production from methanol was investigated by using an Inconel fixed-bed reactor (PID Microactivity, Micromeritics, 9 mm internal diameter). A mixture of methanol/nitrogen (0.016 mol/h of methanol and 0.146 mol/h of N_2_), with a space velocity (WHSV) of 7.55 h^–1^, was fed to the reactor containing 70 mg of the catalyst and 2 g of silicon carbide (SiC) (mesh 200–400) with a total flow rate of 50 mL/min. Catalytic tests were carried out in the 120–200 °C range under atmospheric pressure. Reactor outstream composition was analyzed by an online gas-chromatograph (Shimadzu 2010) equipped with a flame ionization detector (FID).

Methanol conversion was calculated as follows:
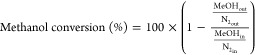
1where MeOH_in_ and N_2_in__ refer to the methanol and nitrogen molar flows entering the reactor and MeOH_out_ and N_2out_ the outlet molar flows.

The selectivity was calculated as
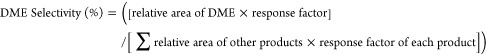
2

### 2.4 Characterization Methods

Powder X-ray diffraction analysis was conducted using a Rigaku Miniflex II (Cu Kα radiation) in 2θ ranging from 5° to 55°. A commercial FER sample was used as a reference (100% crystallinity) for calculating the crystallinity percentage of the interconverted zeolites, based on the diffraction peaks at 2θ = 24.8° and 25.4°.^29^

The morphology of the materials was analyzed by scanning electron microscopy (SEM) using a JEOL model JSM-IT700HR equipped with an X-ray energy dispersive detector (EDS) and transmission electron microscopy (TEM) using a JEOL model JEM-2100F. EDS elemental analyses were performed in order to obtain the bulk Si/Al ratio. The Raman spectra were recorded on an Xplora/Horiba dispersive Raman system with a laser source of 532 nm.

The acidity of the zeolites was determined via temperature-programmed desorption of ammonia (NH_3-_TPD) using the Micromeritics AutoChem II Chemisorption Analyzer equipped with a thermal conductivity detector (TCD). Zeolite samples (100 mg) were pretreated at 300 °C for 1 h under a helium flow (25 mL/min). Subsequently, ammonia chemisorption (15% in helium) was carried out at 150 °C for 1 h. The desorption of NH_3_ was then monitored as the temperature ramped up at a rate of 10 °C/min until it reached 500 °C.

Magic angle spinning nuclear magnetic resonance (MAS NMR) was conducted to quantitatively calculate the sample framework Si/Al ratio. This method was performed on a Bruker Avance III 400WB spectrometer, operating at 104.23 MHz for ^27^Al with a single pulse and a rotation rate of 12 kHz and at 79.46 MHz for ^29^Si with a single pulse and a spin rate of 5 kHz.

The textural characterization of the materials was carried out using an Autosorb iQ (Anton-Paar, Graz, Austria) surface area and porosity analyzer. The samples were degassed at 250 °C for 4 h under high vacuum. The surface area was determined using the Brunauer–Emmett–Teller (BET) method, while the microporous and mesoporous volume was determined using NL-DFT.^30^

## 3 Results and Discussion

### 3.1 Characterization

The samples, prepared as aforementioned, were recovered at various interconversion times and meticulously characterized to gain insights into the evolution of their properties throughout the interconversion process. For comparison purposes, we include in this study both the parent Y zeolite (CBV712) and a commercial FER zeolite (H-FER).

**Figure 1:**
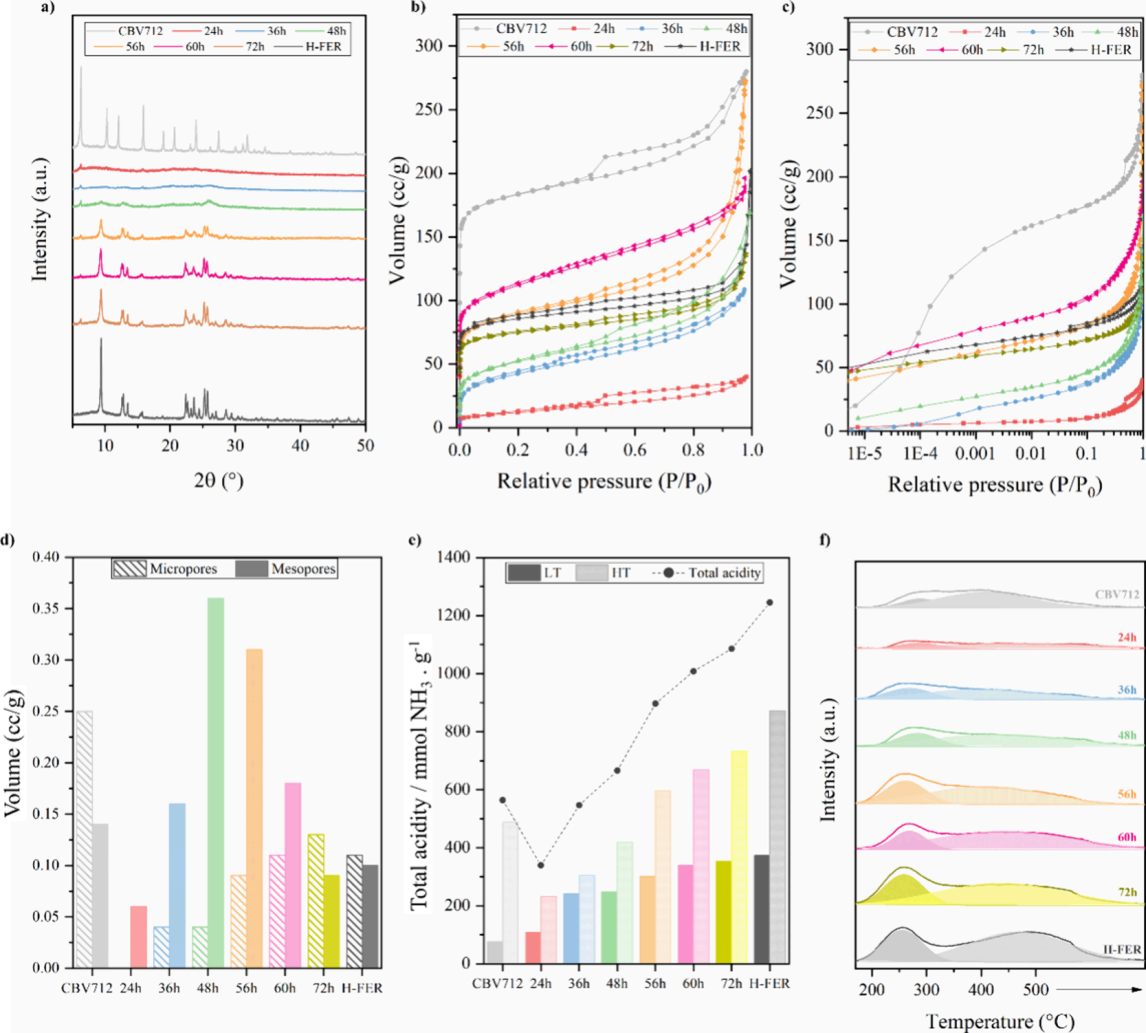
Characterizations of the catalysts: (a) XRD patterns, (b) N_2_ physisorption, (c) N_2_ physisorption plotted in a logarithmic scale to better show their microporosity, (d) micro- and mesopore volume, (e) NH_3_-TPD, and (f) total acidity per gram of zeolite for the ITIs samples.

Figure 1a shows the X-ray diffraction (XRD) patterns of the FAU-to-FER ITIs produced in this study as a function of time. The crystalline structure of the parent FAU zeolite is rapidly lost, as previously described.^23^ After 24 h of treatment, the material was amorphous, but soon after (36 h), the peaks characteristic of the FER structure started to develop. Already after 56 h, 51% of the total crystallinity was obtained. Four hours later, the crystallinity was already more than 91%. To achieve the same level of crystallinity using conventional autoclave heating would require 192 h.^25^ As we will describe later, the ITIs materials, including those showing no X-ray diffraction peaks, contain building units of both FAU and FER zeolites, as confirmed by Raman and ^29^Si NMR spectroscopies (Figure 3).

The evolution of the textural properties of the samples produced during the interzeolite conversion process was monitored by N_2_ physisorption (Figure 1b). The initial sample, obtained after 24 h, exhibits almost no micropore volume, which agrees with the low crystallinity and low acidity of this material (Table 1 and Figure 1a–f). After 36 h, some microporosity starts to develop, and this trend continues with treatment time, reaching a maximum micropore volume of 0.13 cc/g for the 72 h sample which is even slightly higher than the commercial sample (H-FER). On the other hand, the mesoporosity shows a volcano-like behavior with a maximum mesopore volume of 0.36 after 48 h sample of treatment (Figure 1c and Table 1).^23,24^

**Table 1:** Textural and Acidic Properties of the Samples^a^

	crystallinity^b^ (%)	framework Si/Al^c^	bulk Si/Al^d^	*S*_BET_^e^ (m^2^/g)	*V*_micro_^f^ (cc/g)	*V*_meso_^f^ (cc/g)	LT^g^ (μmol/g_cat_)	HT^g^ (μmol/g_cat_)	total acidity^g^ (μmol/g_cat_)
CBV712		6.0	6.3	800	0.25	0.14	75	489	564
24 h	15	4.6	7.9	53	0.00	0.06	108	231	339
36 h	19	5.7	7.8	218	0.04	0.16	241	305	546
48 h	25	5.8	7.9	233	0.04	0.36	248	418	666
56 h	51	8.5	7.4	329	0.09	0.31	301	596	897
60 h	91	8.9	7.6	336	0.11	0.18	340	668	1008
72 h	93	8.9	7.7	407	0.13	0.09	353	733	1086
H-FER	100	10.0	9.9	326	0.11	0.10	373	872	1245

a Crystallinity, Si/Al ratio, and main textural and acidic properties of interconverted samples produced at different time intervals. Both FAU (CBV712) and FER (H-FER) commercial samples are also listed as reference materials.

b Percentage of crystallinity estimated by XRD.

c Framework Si/Al ratio obtained by ^27^Al and ^29^Si NMR analyses.

d Bulk Si/Al ratio obtained by EDS elemental analysis.

e Specific surface area calculated using the BET method.

f Volume of micropores and mesopores calculated using the NL-DFT model.

g Total acidity was calculated using NH_3_-TPD.

A key observation that is crucial for understanding the unique catalytic performance of these ITIs, is that after 56 h of treatment, all ITIs exhibit very similar N_2_ uptake behavior at 77 K at low relative pressures, almost identical to that of the H-FER zeolite (Figure 1c). This strongly indicates that these materials share a similar confinement effect, which, as discussed later, significantly impacts the selectivity of these catalysts in various reactions.

**Figure 2:**
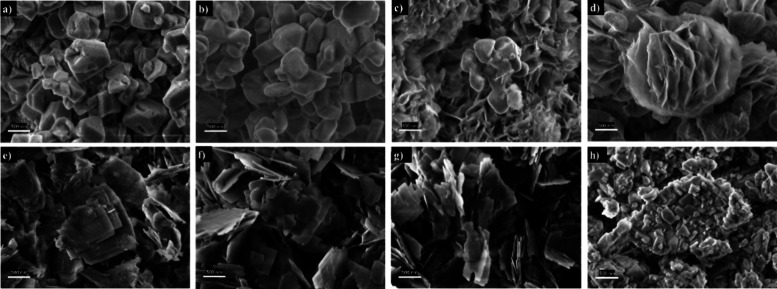
Scanning electron microscopy images. (a) CBV712, (b) 24 h, (c) 36 h, (d) 48 h, (e) 56 h, (f) 60 h, (g) 72 h, and (h) H-FER. Transmission electron microscopy images of (i) 48 h, (j) 56 h, and (k) 60 h.

The morphological evolution of the samples was monitored using both scanning and transmission electron microscopies (SEM and TEM). Representative micrographs are displayed in Figure 2. During the initial phases of the interconversion (24 and 36 h), the morphology resembled that of the precursor, zeolite Y (Figure 2a–c). Interestingly, the 48 h sample marks a steep transition in morphology, being the first sample to exhibit some plates. Samples obtained after 56, 60, and 72 h of treatment all show a plate-like morphology, (Figure 2e–g) akin to ferrierite used as the reference material, as widely reported elsewhere.^31^

On the other hand, the TEM micrographs show that the sample obtained after 48 h of treatment (Supplementary Figure 1) displays large voids, which could be related to the dissolution of the FAU zeolite precursors. This morphology is even more prominent in the sample produced after 56 h (Supporting Information, Figure 2). Only 4 h later, the presence of well-formed plates is quite evident (Supplementary Figure 3) as expected due to the well-defined XRD peaks and high micropore volume of this sample (Figure 1a–d).

**Figure 3:**
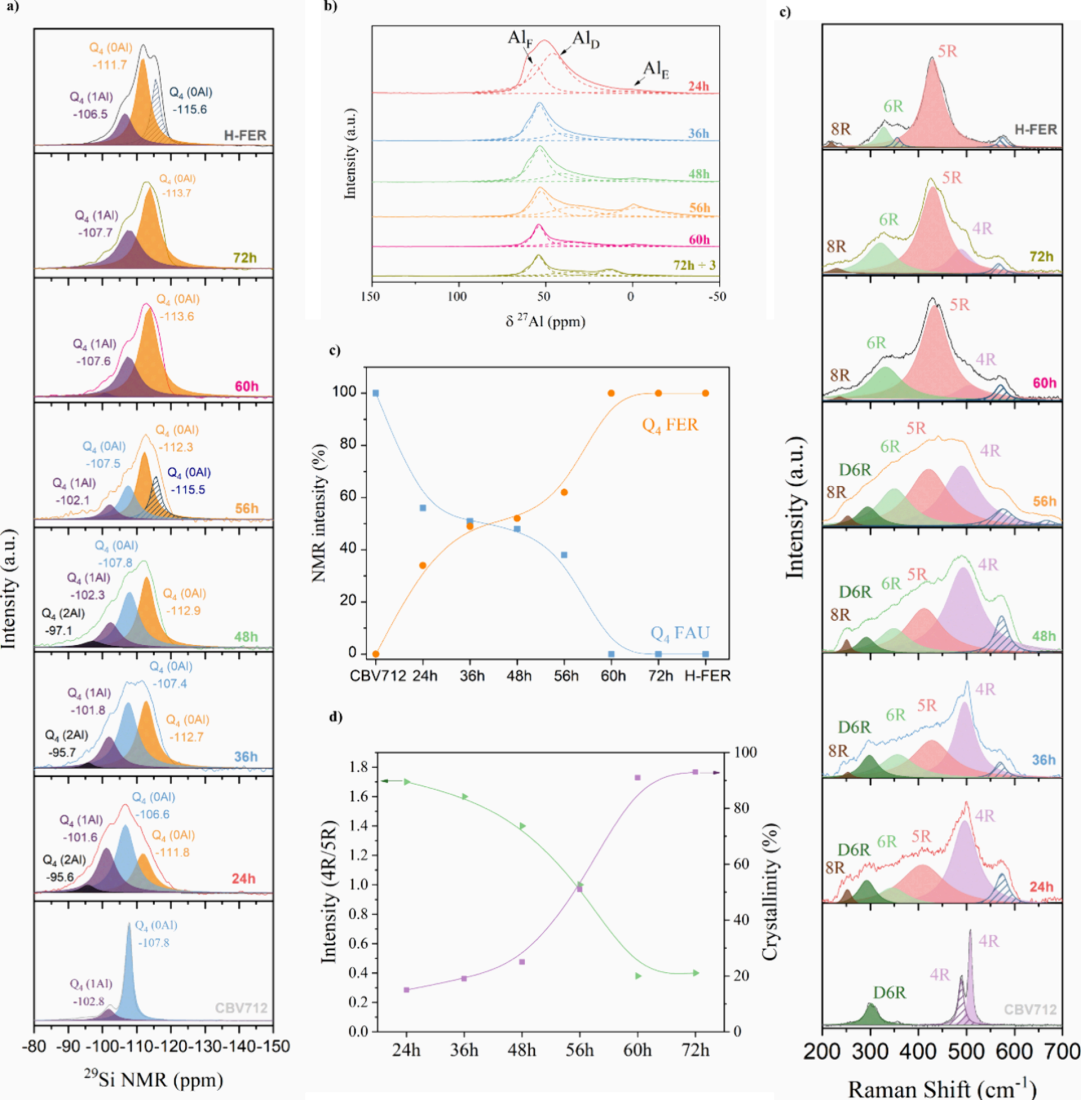
Raman and RMN analyses of the interconverted samples. (a) ^29^Si NMR and (b) ^21^Al NMR spectra of the interconverted and reference samples. (c) Evolution of the percentage of Q_4_ (0Al) intensity in ^29^Si NMR of the FAU and FER zeolite. (d) Evolution of the 4R/5R ratio of the samples, as determined by Raman spectroscopy as a function of the percentage of crystallinity as calculated by XDR. (e) Raman spectra of the interconverted samples. Each spectrum was deconvoluted to allow for the monitoring of the evolution of the various building units in the ITIs.

In order to gain new insight into the structural changes that occur during the interconversion process, spectroscopic characterizations were carried out. The results are shown in Figure 3 and Table 1. Consistently, the bulk Si/Al ratio was similar to those of all ITIs. The evolution of the framework Si/Al ratio throughout the interconversion process was followed by ^27^Al and ^29^Si NMR analyses (Figure 3a,b). As expected, the most amorphous material (24 h) has the lowest Si/Al ratio among all the ITI samples (see Table 1), which is related to the partial dissolution of the parent FAU zeolite. However, as the interconversion progresses, the framework Si/Al ratio rises until it reached a value close to that of the H-FER zeolite. Figure 3a,b depicts the ^27^Al and ^29^Si NMR spectra for all the ITIs produced, in addition to the two reference materials, the parent and the daughter zeolites. In terms of structural Al composition (Figure 3b), the deconvoluted peaks centered at 54–61 ppm (Al_F_), 44–45 ppm (Al_D_), and 0 ppm (Al_E_) correspond to framework Al, distorted framework Al, and extra-framework Al (EFAl), respectively.^32−34^ It is evident that with increasing crystallinity, the intensity of distorted Al peaks diminishes, while framework Al peaks intensify. During the interconversion process from FAU to FER, the deconstruction of the FAU structure promotes the formation of extra-framework aluminum, whose amount increases as the interconversion time progresses (Figure 5b). This phenomenon was previously reported^25^ and can be associated with the difference in the usual Si/Al ratio between the two zeolites, with FAU exhibiting a lower Si/Al ratio than FER.

Valuable insights into the hybrid nature of the ITIs were obtained from ^29^Si NMR spectra (Figure 3a). In the FAU spectrum, two main peaks are observed: one at −107.6 ppm corresponding to Q4 (0Al) and another at −101.7 ppm corresponding to Q_4_ (1Al).^24,35^ In the FER spectrum, two peaks were observed, with the maxima at −111.7 and −115.6 ppm which are assigned to Q_4_ (0Al), and a small peak at −106.5 ppm which may be assigned to Q_4_ (1Al). In the ITIs peaks corresponding to FAU and FER zeolites were not found at the same resolution because of the disorder around each silicon atom created by the substitution of aluminum into the framework.^34,36^ Henceforward, we decided to analyze the interconversion process by monitoring the evolution of the chemical shift of the band associated with Q_4_ (0Al) for both structures. As shown in Figure 3c, throughout the interconversion, the Q_4_ (0Al) band in FAU became less intense, while the Q_4_ (0Al) band in FER developed. Also, as the Q_4_ (0Al) increases in intensity (−107.6 ppm in FAU), it shifts toward the value of the Q_4_ (0Al) in FER zeolite, which is between −111.7 and 115.6 ppm, until it fades out completely (Figure 3a). These observations show the hybrid character of the ITIs samples and how by stopping the interconversion of different times we can produce materials with continuous structural compositions between the parent and the daughter zeolites. It is worth mentioning that the short times of interconversion (24, 36, and 48 h), which present a lower Si/Al ratio, bands at −95.6, −95. 7, and 97.1 ppm, can be observed. Such bands, related to Q_4_ (2Al), may indicate the formation of the six-membered rings of the FER zeolite in low Si/Al ratio samples.^34,37^

The structural evolution of the samples was also analyzed by Raman spectroscopy.^38^ As shown in Figure 3e, both the parent FAU zeolite and the daughter FER zeolite show very distinctive bands, which are associated with their respective building units^24,39,40^ More specifically, the Raman spectrum of the crystalline FAU zeolite structure presents three main bands at (I) at 300 cm^–1^, related to the double six-membered ring (D6R);^41^ (II) at 490 cm^–1^, related to the 4-membered ring (4R) containing D6R;^39^ and (III) at 503 cm^–1^, related to the 4R.^32^ During the interconversion, the band associated with the D6R unit progressively decreases. Simultaneously, the band corresponding to the 4R unit undergoes a slight shift and gradually diminishes. Meanwhile, the 5R unit is generated, leading to an increase in crystallinity, as shown in Figure 3d. The presence of the 4R unit in the ITIs indicates that not all of the material is dissolved during the interconversion process. As treatment time increases, the characteristic bands of FER zeolite at 218 cm^–1^ (8R), 315 cm^–1^ (6R), and 430 cm^–1^ (5R) begin to develop.^40^ This implies the transformation of the D6R unit into 6R units rather than 4R.^39^ Additionally, the formation of the 8R unit occurs gradually, showing shifts during the treatment, as shown in Figure 3e.

Our findings indicate that during the interconversion from FAU to FER zeolites, intermediates are initially formed from an amorphous material.^42^ XRD analyses showed that this precursor lacks a distinct crystalline structure. However, Raman spectroscopy analyses suggest the remaining presence of structural units of the precursor material in the ITIs,^43^ indicating that the dissolution of the initial material is incomplete. This observation is corroborated by both the SEM analysis and the initial reduction observed in the Si:Al ratio values, which point to a process of partial dissolution of the precursor zeolite. In addition, the N_2_ physisorption data highlights a significant increase in the volume of mesopores in the ITIs, which greatly contributes to enhancing the accessibility to their active sites, which is crucially important, as we will see in the enhancement of their catalytic performance.^44^

### 3.2 Catalytic Tests

We selected three different acid-catalyzed reactions, namely, the Friedel–Crafts alkylation of benzyl alcohol with mesitylene, the cracking of triisopropylbenzene, and the production of dimethyl ether from methanol, to evaluate not only the strong acidity of these materials but also their increased diffusion properties, which are required to convert bulky molecules and reduce deactivation due to coke formation.

#### 3.2.1 Friedel–Crafts Alkylation (FCA)

We chose the Friedel–Crafts alkylation of benzyl alcohol (BA) with mesitylene (ME) as a test reaction to assess both the accessibility and the acidity of our catalysts.^11,45^ More specifically, for ferrierite this reaction mainly occurs on its external surface area because neither mesitylene (kinetic diameter = 0.84 nm)^46^ nor benzyl alcohol (kinetic diameter = 0.58 nm)^47^ can diffuse well into the narrow micropores of this topology.^47^ On the other hand, in the case of faujasite, BA can easily access the micropores, leading to the production of dibenzyl ether (DBE).

**Figure 4:**
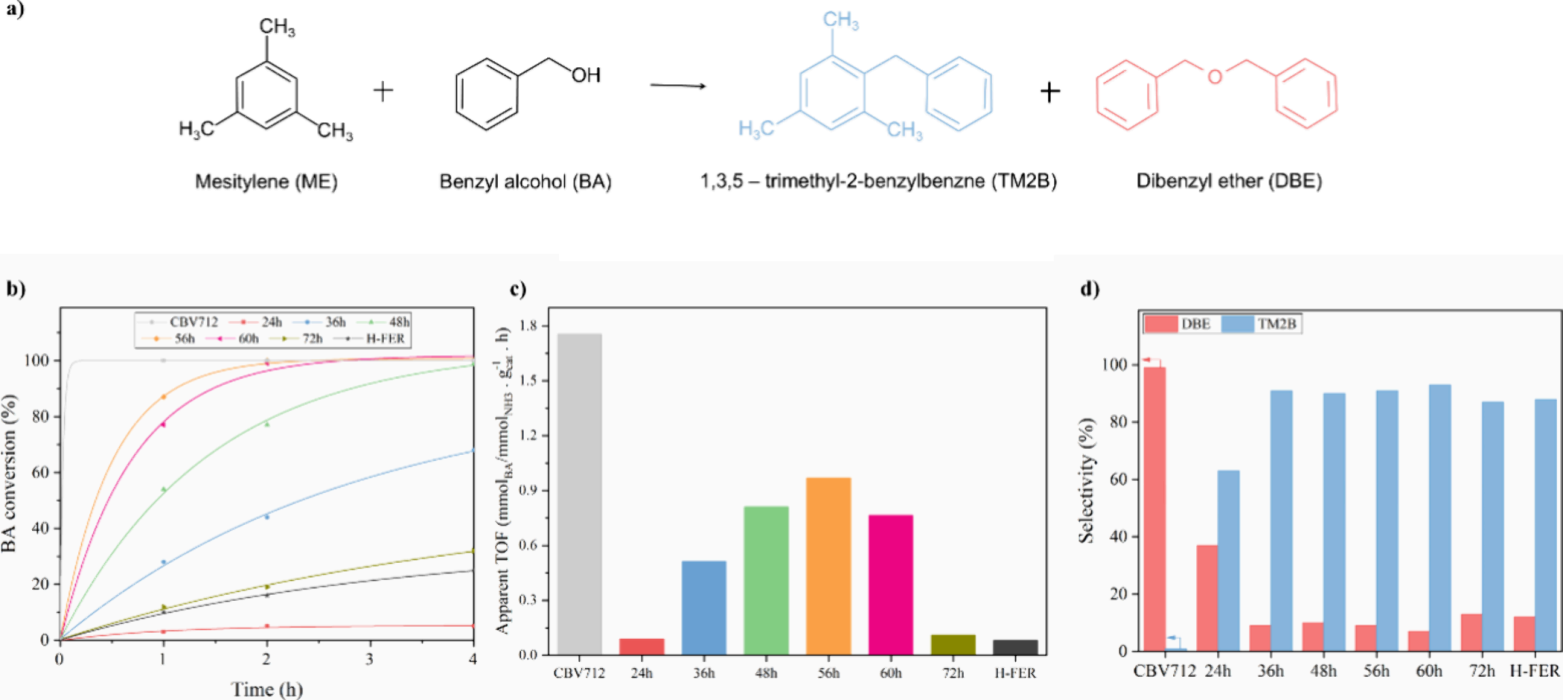
Catalytic performance of the catalyst in the Friedel–Crafts alkylation of mesitylene and benzyl alcohol. (a) Friedel–Crafts reaction scheme: mesitylene reacts with benzyl alcohol to produce 1.3.5-trimethyl-2-benzylbenzene (TM2B) and dibenzyl ether (DBE). (b) Conversion profiles of benzyl alcohol in Friedel–Crafts alkylation by the interconverted zeolites as a function of time, (c) apparent TOF, and (d) selectivities to either TM2B or DBE measured at an isoconversion of 10%.

In terms of conversion, all ITIs, except that prepared at 24 h, outperformed the commercial zeolite (H-FER), see Figure 4b. This is even more clear in Figure 4c which shows a volcano shape for the apparent TOF of all the samples, including the reference materials, with a maximum for the ITI prepared at 56 h. This sample shows a remarkable 10-fold increase in TOF compared to that of the commercial FER sample. This superior performance can be explained by a combination of enhanced accessibility and acidity.^24^ This conclusion is confirmed by the abrupt decrease in catalytic activity after 72 h of treatment, as this sample is already very similar to a fully formed FER zeolite, with negligible mesoporosity, confirming that shape selectivity in a fully crystalline FER led to lower conversions due to poor accessibility of the bulky reactants. Supplementary Figure S4 demonstrates the behavior of both the ratio between strong and weak acid sites (HT/LT) and the apparent TOF for each material. It is noteworthy that the HT/LT ratio increases with the interconversion duration, while the TOF starts to decrease for samples 60 h and beyond, further confirming that the acidity strength or acid site type is not solely responsible for the catalytic activity. Obviously, the most active material is the FAU zeolite (CBV712), as its wider micropores allow full access to BA (the limiting reactant), resulting in fast self-consumption of this compound into DBE.

It is remarkable that all ITIs, except the one obtained at 24 h, which according to our data still possesses more FAU characteristics, exhibit identical selectivity to commercial FER. This suggests that the pore confinement of the FER, which is responsible for excluding both BA and ME from accessing the microporous matrix, is preserved in the ITIs. Even with catalysts, a 10-fold increase in the conversion was observed for this reaction.

#### 3.2.2 Cracking of Triisopropylbenzene (TiPBz)

The FCA test results showed that the ITIs produced after 56 h of treatment exhibited remarkably superior catalytic performance owing to the combined presence of high mesoporosity and strong acidity. Moreover, the test showed that this material, despite the apparent disordered structure has characteristics of both FER and FAU topologies, yielded similar selectivities to the commercial FER in the FCA.

To gain more insights into this unusual catalytic hybrid behavior of combining the high activity of FAU together with shape selectivity derived from FER was investigated. We decided to test our best ITI (56 h) against the parent Y zeolite (CBV712) and the commercial FER (H-FER) in TiPBz. This molecule, with a kinetic diameter of 0.94–0.95 nm,^48^ is unable to penetrate the micropores of either FER or Y zeolites so the first cracking should only occurs in external surface of all the samples.

**Figure 5:**
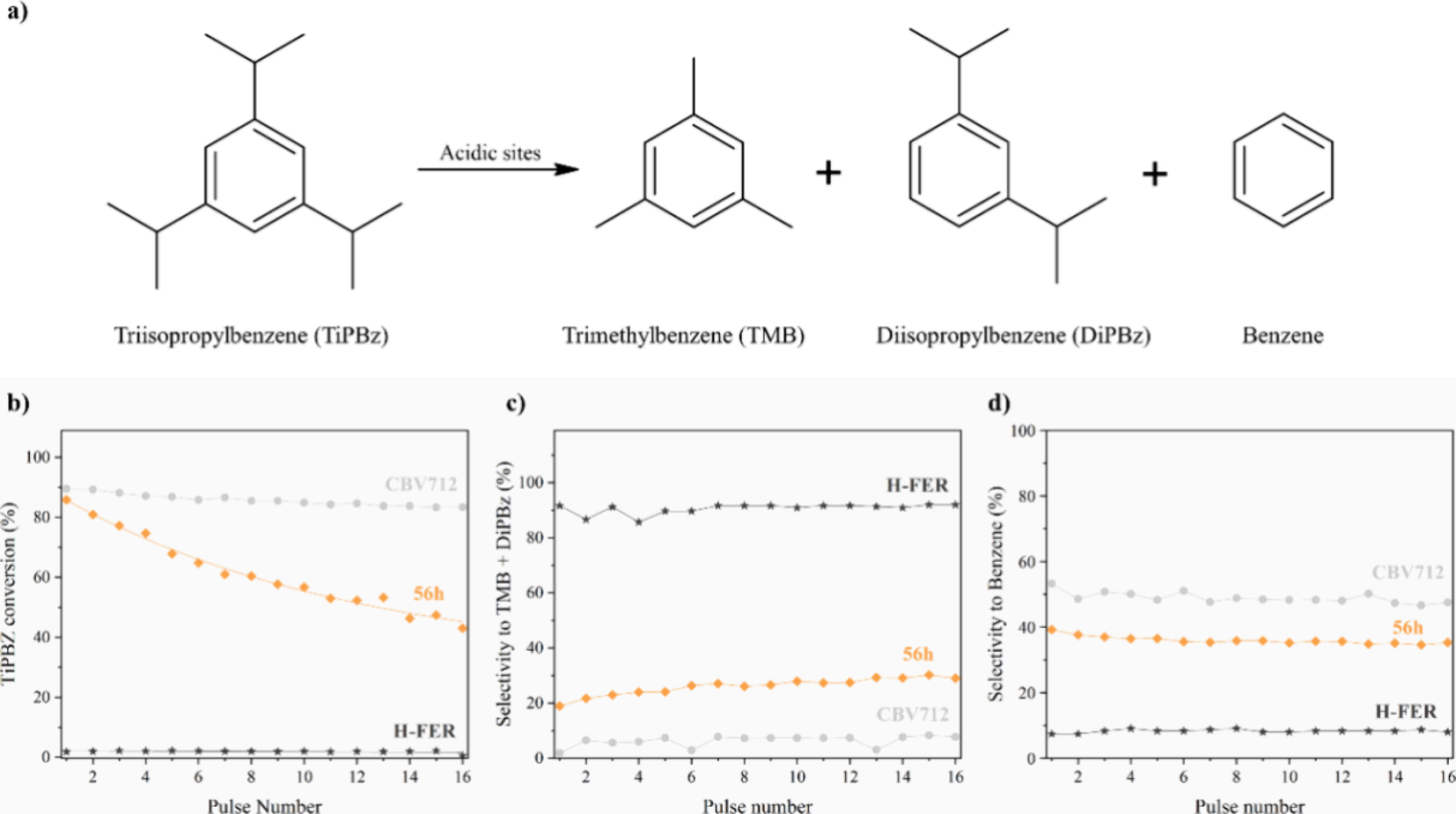
Catalytic performance of 56 h, CBV712, and H-FER samples in the cracking of triisopropylbenzene (TiPBz). (a) Reaction scheme for TiPBz cracking. (b) TiPBz conversion profiles at 220 °C. Selectivity to (c) trimethylbenzene (TMB) and mono dealkylation product (DiPBz), and (d) benzene.

Figure 5b shows the conversion profile of TiPBz as a function of the number of pulses. There is a significant difference in conversion levels between the ITI prepared after 56 h of treatment and the commercial H-FER. Notably, the catalytic activity of this ITI in the initial pulses closely resembles that of the parent Y zeolite (CBV712) rather than the fully crystallized FER zeolite. However, in terms of selectivity, in this case, the sample exhibits a hybrid nature. It shows intermediate selectivity between H-FER and Y zeolites for both the full dealkylation product (benzene) and the mono dealkylation product (DiPBz) and trimethylbenzene (TMB) (see Figure 5c,d). This observation again indicates that ITI retains some shape selectivity associated with the FER structure, which is very interesting and confirms the presence of FER building units (local order) and FER-type microporosity in this material (see Figure 1c).

#### 3.2.3 DME Production via Methanol Dehydration

Methanol dehydration to DME is a highly relevant reaction in the context of the energy transition, as DME is often used as an intermediate in the production of both fuels and chemicals from CO_2_.^49,50^ This reaction is highly sensitive to the zeolite structure, with FER standing out as one of the best-performing zeolites. Its 2D structure with medium-sized channels results in a catalyst that offers excellent selectivity, even at higher conversion rates. The loss of both the activity and selectivity of the zeolites in this reaction was also highly affected by both inter- and intracrystalline mesoporosities.^3^

**Figure 6:**
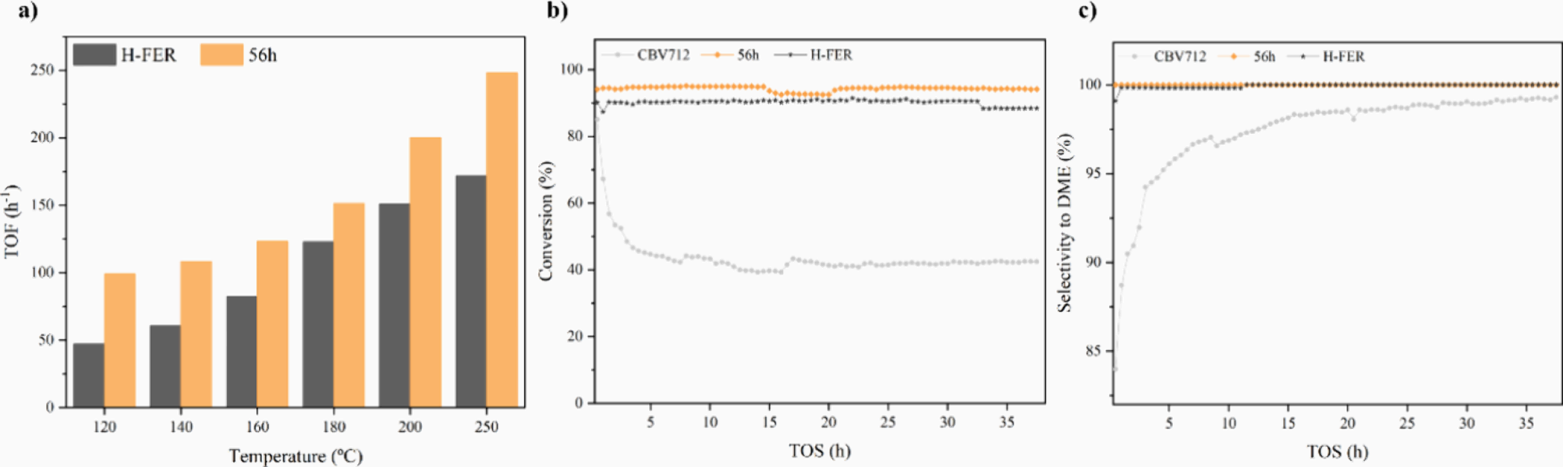
Catalytic performance of the 56 h and H-FER samples in DME production via methanol dehydration. TOF of methanol to the DME reaction by the 56 h interconverted zeolite and the H-FER (a), conversion profiles of methanol in methanol to DME reaction at 250 °C for the parental zeolite CBV712, the ITI 56 h, and the H-FER (b), and selectivities to DME at 250 °C for the parental zeolite CBV712, the ITI 56 h, and the H-FER (c).

To overcome this mutually exclusive limitation, we tested our best-performing catalyst (obtained after 56 h of treatment) against a commercial FER (H-FER). Our goal was to enhance the conversion by introducing mesoporosity—thereby improving the diffusion properties of this medium-pore-size zeolite—while preserving the confinement effect of the FER, which is responsible for its excellent selectivity and stability in this reaction. As expected, the H-FER zeolite was greatly outperformed by ITI, which at the lower end of our temperature test range confirms that increasing accessibility results in higher conversion. As the temperature increased, the kinetics improved, causing the conversion rates of both systems to converge. When the results are plotted in terms of the TOF, the differences become more striking. The ITI shows a TOF that is 30% higher than that of the H-FER even at the higher end of our temperature range. Unexpectedly, but consistent with the aforementioned observations the Y zeolite (CBV712) has a half-life of only 4 h, whereas the ITI and H-FER samples virtually do not deactivate even after 38 h under the reactions conditions. This new observation confirms that the ITI obtained at 56 h behaves similarly to H-FER in terms of not only selectivity but also in terms of deactivation. Both catalysts (H-FER and 56 h) exclusively produce DME (see Figure 5c), indicating that the increase in activity observed for the ITI does not come at the expense of selectivity. In the case of FAU, despite its high initial activity, its more open structure reduces the selectivity by allowing the formation of other products (see Supporting Information Figure 7). This observation further supports the preservation of the confinement effect that we mentioned before in the ITIs (Figure 6).

## 4 Conclusions

This study explored the catalytic performance of interzeolite transformation intermediates (ITIs) derived from FAU-to-FER interconversion. These materials exhibit a unique combination of high mesoporosity and FER topology-type pore confinement. This leads to a remarkable increase in the catalytic conversion of bulky molecules, demonstrating the enhanced accessibility of these materials along with exceptional shape selectivity.

More specifically, the ITI sample obtained after 56 h of treatment exhibits a 10-fold increase in activity for Friedel–Crafts alkylation, a 16-fold rise in triisopropylbenzene cracking, and a 2-fold improvement in methanol dehydration to dimethyl ether (DME), all compared to commercial ferrierite. These improvements were achieved without compromising the inherent selectivity and stability of FER zeolites, highlighting the practical utility of these ITIs.

A key aspect of this enhanced performance is the unique structural arrangement of the ITIs, which comprise building units of both FAU and FER topologies combined with the increased accessibility afforded by mesoporosity. This synergy results in more active catalysts that maintain selectivity and stability in reactions in which shape selectivity is crucial, such as in DME production. Notably, in the production of DME, these ITIs not only enhance activity but also exhibit remarkable stability, resisting deactivation over extended periods. In conclusion, this study introduces a novel methodology for preparing interzeolite transformation intermediates through FAU-to-FER interconversion. For the first time, we demonstrate with catalytic data from three distinct reactions that these materials successfully combine the best features of FER topology-type pore confinement and large mesoporosity. By halting the interzeolite transformation at strategic times, we took advantage of the unique properties of local order and mesoporosity, achieving an optimal balance between accessibility and pore confinement.

## Supporting Information Available

TEM images of the materials and more information about the catalyst tests (PDF)
