# Supplementary material for: How Local Order Leads to Shape Selectivity in Disordered Materials: The Case of FAU-FER Interzeolite Transformation Intermediates
Source: ACS Catal. 2025 Mar 4;15(6):4586–95. doi: 10.1021/acscatal.4c07182 (PMC11934296; doi:10.1021/acscatal.4c07182)
Supplement: Supplementary file 1 — cs4c07182_si_001.pdf [file cs4c07182_si_001.pdf]

Supporting Information

How local order leads to shape-selectivity in disordered materials: The case of FAU-FER Interzeolite Transformation Intermediates

Julia T. de Souza[a,b], Alexandre F. Young[a,b], Eduardo F. Sousa-Aguiar[a,b], Pedro N. Romano[b,c,d], Javier Garcia Martinez[e,] and João M.A.R. de Almeida[b,f,*]*

[a] Escola de Química, Universidade Federal do Rio de Janeiro, Av. Athos da Silveira Ramos, 149, Rio de Janeiro, Brazil, 21941-909.

[b] LIPCAT (Laboratório de Intensificação de Processos e Catálise), Universidade Federal do Rio de Janeiro (UFRJ), Rio de Janeiro 21941-594, RJ, Brazil.

[c] Campus Duque de Caxias, Universidade Federal do Rio de Janeiro, Rodovia Washington Luiz, 19593, Rio de Janeiro, Brazil, 25240-005.

[d] Nanotechnology Engineering Program, Alberto Luiz Coimbra Institute for Graduate Studies and Research in Engineering (COPPE), Federal University of Rio de Janeiro, Avenida Horacio Macedo, 2030 21941-972, Rio de Janeiro, RJ, Brazil

[e] Laboratorio de Nanotecnología Molecular, Departamento de Química Inorgánica, Universidad de Alicante, 03690, Alicante, Spain. ORCID:0000-0002-7089-4973

[f] Instituto de Química, Universidade Federal do Rio de Janeiro, Av. Athos da Silveira Ramos, 149, Rio de Janeiro, Brazil, 21941-909.

*j.garcia@ua.es (J. García-Martínez), *j.monnerat@iq.ufrj.br (J.M.A.R. de Almeida)

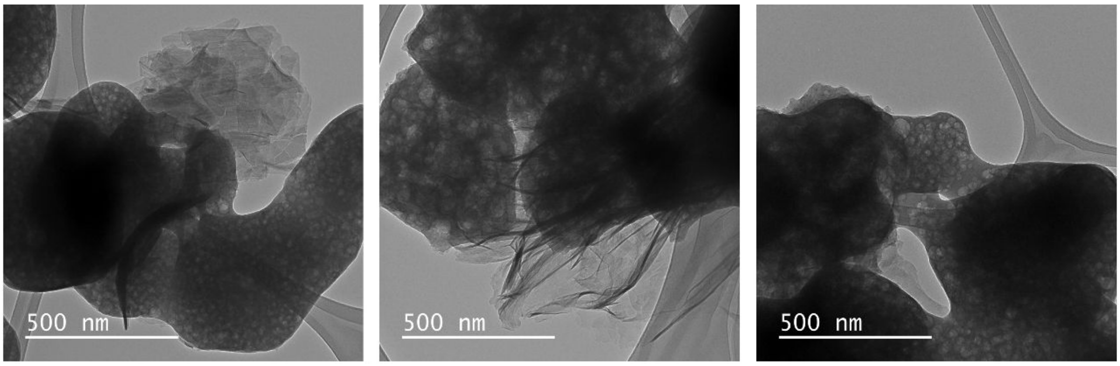

Figure S1. TEM imagens of 48h interconverted zeolite

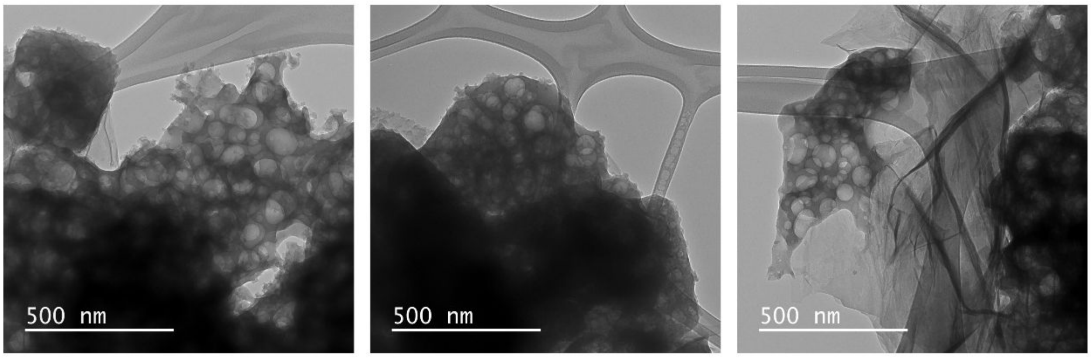

Figure S2. TEM imagens of 56h interconverted zeolite.

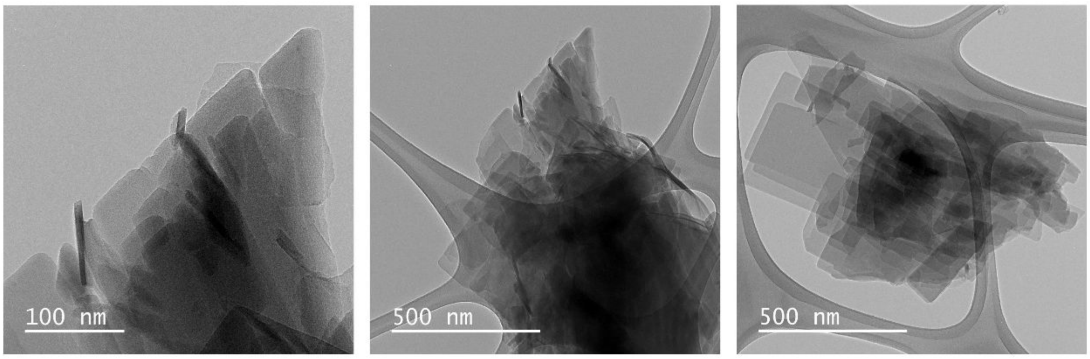

Figure S3. TEM imagens of 60h interconverted zeolite

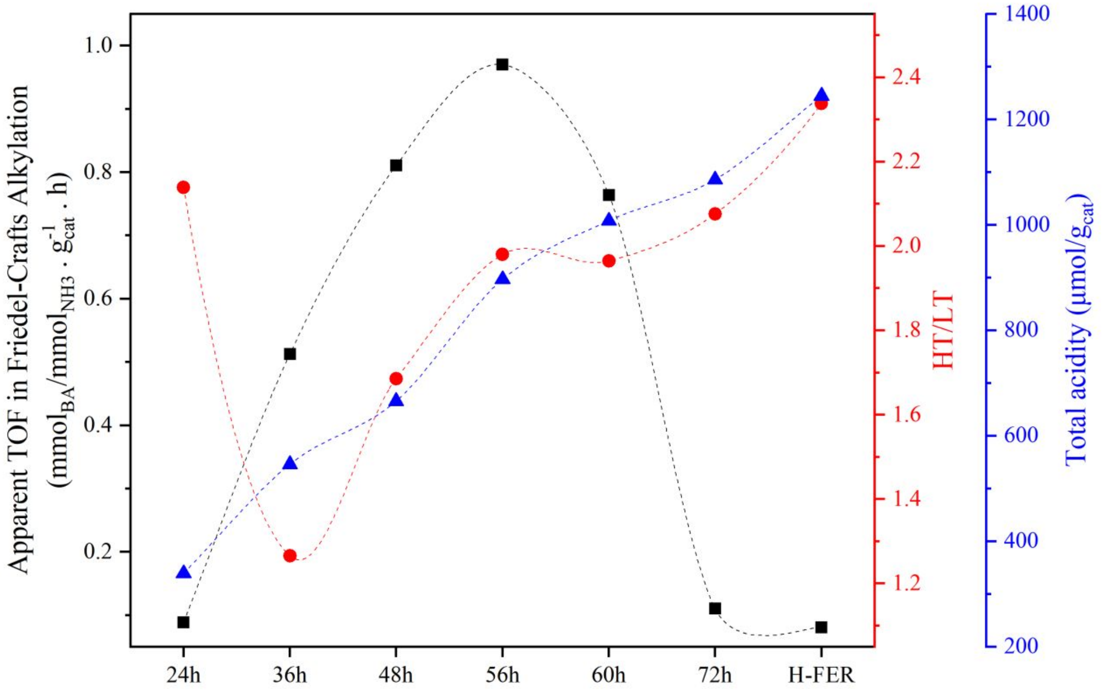

Figure S4. Correlation between apparent TOF in Friedel-Crafts alkylation (left axis; black), strong and weak acid sites (right axis; red) and total acidity (left axis; blue) for each ITI and for H-FER reference material.

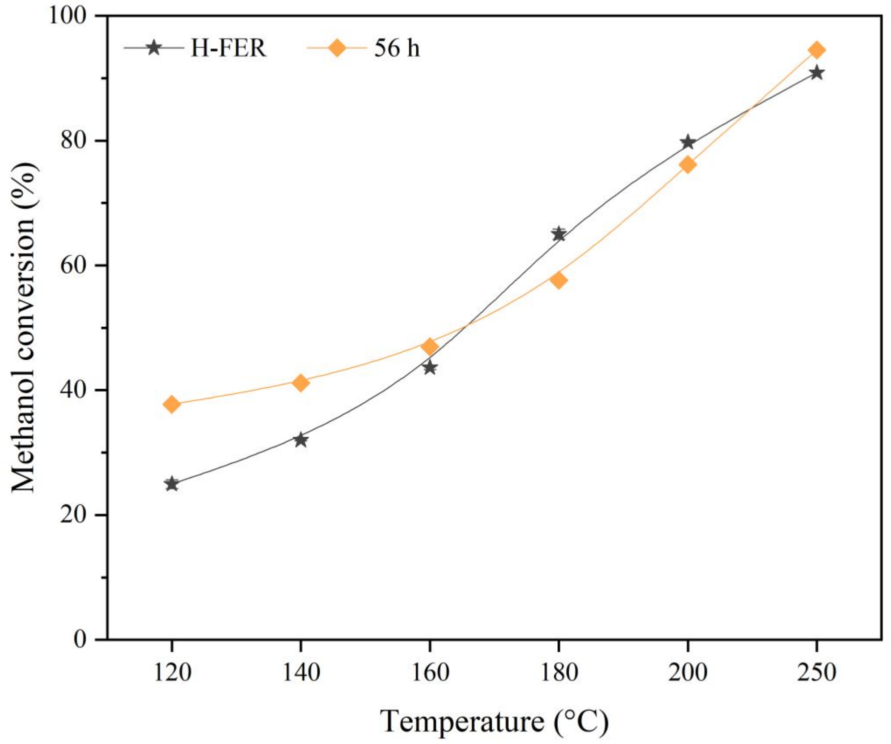

Figure S5. Conversion profiles of methanol in methanol to DME reaction by the 56h interconverted zeolite and the H-FER

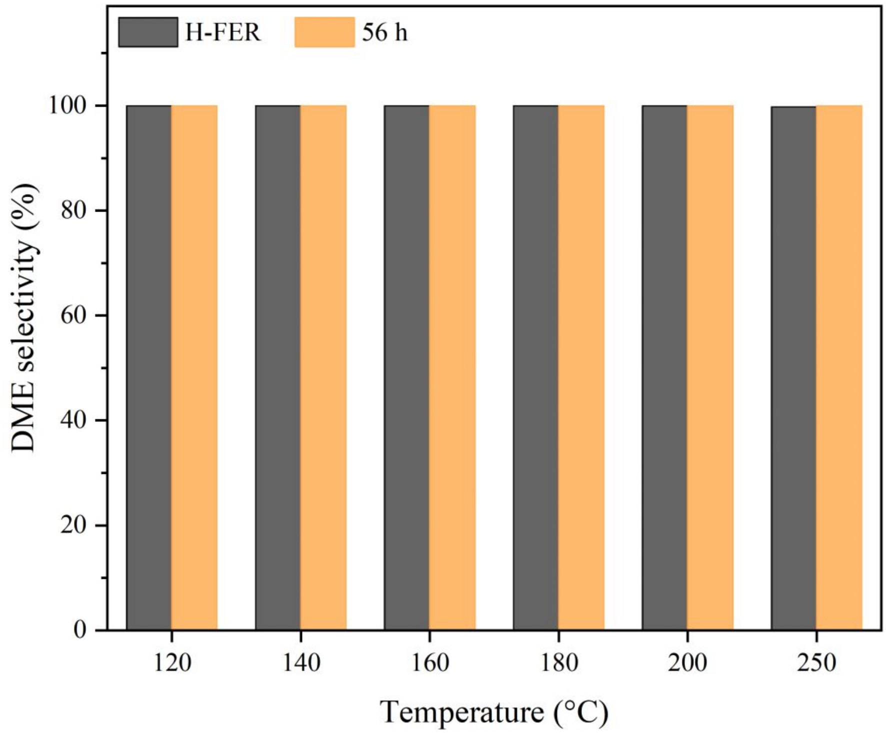

Figure S6. Selectivities to DME by the 56h interconverted zeolite and the H-FER

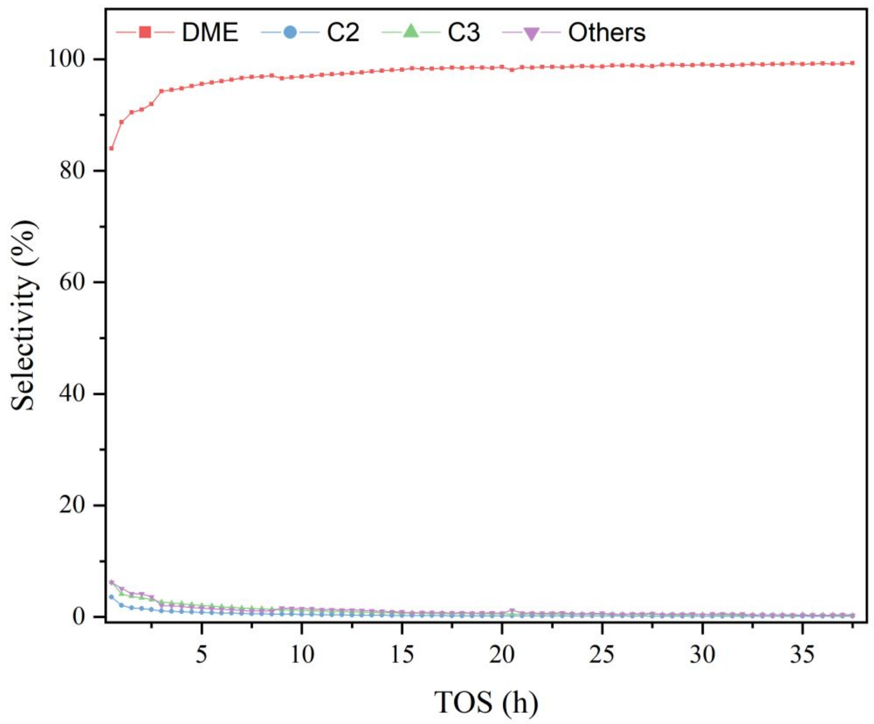

Figure S7. Selectivities to DME at 250 °C by the parental zeolite CBV712.
